# Probiotic effect of *Pichia pastoris* X-33 produced in parboiled rice effluent and YPD medium on broiler chickens

**DOI:** 10.1371/journal.pone.0192904

**Published:** 2018-02-15

**Authors:** Diego Gil de los Santos, João Rodrigo Gil de los Santos, Carlos Gil-Turnes, Giana Gaboardi, Luiza Fernandes Silva, Rodrigo França, Cristina Gevehr Fernandes, Fabricio Rochedo Conceição

**Affiliations:** 1 Centro de Desenvolvimento Tecnológico, Universidade Federal de Pelotas, Pelotas, RS, Brazil; 2 Instituto Federal Sul-riograndense, Pelotas, RS, Brazil; 3 Faculdade de Veterinária, Universidade Federal de Pelotas, Pelotas, RS, Brazil; Maharshi Dayanand University, INDIA

## Abstract

In a previous paper we showed that the yeast *Pichia pastoris* X-33 grown in parboiled rice effluent supplemented with glycerol byproduct from the biodiesel industry improved the quality of the effluent. In this paper we show the validation of this yeast (PPE) as probiotic for broilers. Its effect on feed efficiency and immunomodulation was compared with the same yeast grown in yeast peptone dextrose medium (PPY), with Saccharomyces *boulardii* (SBY) and with the controls fed unsupplemented feed (CON). One-day-old female chicks were vaccinated against infectious bursal disease (IBD) and the titers of anti-IBD antibodies were measured by ELISA. PPE group had the highest mean titres on days 14 and 28 (p<0,05), and at 28 days, 64% of the animals showed seroconvertion. The PPE group also showed the best weight gains at 42 days of age, that, on days 7, 14 and 21 were 19%, 15%, and 8.7% higher, respectively, than the control group. The best feed conversion, 8.2% higher than the control group, was obtained by PPY at 42 days. Histopathological studies did not detect any undesirable effects in the supplemented animals. We concluded that *Pichia pastoris* X-33 when grown in effluents of the rice parboiling industry supplemented with glycerol byproduct from the biodiesel has probiotic properties for poultry.

## Introduction

At the beginning of the century the European Community banned the use of antibiotics as growth promoters or for disease control in animal feeds [1, 2], challenging the broiler industry all around the world to find substitutes for them. Prebiotics, probiotics and symbiotics appeared as alternatives. Several yeasts exhibited prebiotic or probiotic properties improving feed efficiency and enhancing immunity [3–8].

With a world production of 88.7 million tons in 2016, chicken meat is the second highest commodity on the world´s meat production market, being Brazil the world's biggest exporter and the second larger producer [9, 10]. In 2014 the global market for animal feed reached one thousand million tons, 44% of which were for poultry [11].

Residual by-products from agriculture, forestry and the food industry are used as culture media for the industrial production of yeast probiotics [12, 13]. Strains of the genus *Pichia* were grown in waste products of the fermented kimchi industry [14], in an aqueous by-product of the fermentation of lettuce [15], in the residue of cabbage fermentation [16], in waste water from potato chips [17], in a by-product of the biodiesel industry that predominantly contains glycerol [18] and in the effluent of the parboiled rice industry supplemented with a byproduct of the biodiesel industry [19]. *Pichia* sp. NRRL Y-4810 associated with other microorganisms obtained from the residue of the fermentation of black tea [20] and *Pichia guilliermondii*^®^ marketed by CitriStim [21] have also demonstrated probiotic effects. So far the probiotic properties of *P*. *pastoris* grown in effluents of the rice parboiled industry were not studied.

Our group showed that *P*. *pastoris* KM71H has probiotic properties and improves the feed efficiency of broiler chickens [4]. Another study demonstrated that, when grown in an effluent of the parboiled rice industry supplemented with a by-product of the biodiesel industry containing mainly glycerol, *P*. *pastoris* X-33 reduced the effluent’s chemical oxygen demand (COD), nitrogen (N, Kjeldahl Total Nitrogen) and phosphorus contents [19], demonstrating its potential for bioremediation. Furthermore, *P*. *pastoris* X-33 has also demonstrated antibacterial activity against *Salmonella* Typhimurium [22].

In this paper we show the validation of *P*. *pastoris* X-33 grown in an effluent of the parboiling rice industry supplemented with a byproduct of the biodiesel industry as probiotic for broiler chickens in terms of safety, effect on feed efficiency and immunomodulation.

## Materials and methods

All the experiments performed in this study were approved by the Federal University of Pelotas Animal Care Committee in accordance with Brazilian Federal Laws. All the procedures used were approved by the Ethics Committee on Animal Experimentation of the Federal University of Pelotas (process N° 23110.008532/2011-12).

### Animals and housing conditions

One hundred and twenty female one-day-old Cobb 500 chicks purchased from a commercial hatchery (Cooperativa Sul-Rio-Grandense de Laticínios Ltda., Morro Redondo, RS, Brazil) were vaccinated against Infectious Bursal Disease (IBD, Gumboro) and Marek's disease (VECTORMUNE^®^ HVT-IBD, Ceva Saúde Animal Ltda., Paulínia, SP, Brazil). The animals were randomly divided into four groups (30 per group) and housed in 10 m^2^ boxes with concrete floor covered with 10 cm of wood shavings. The animals received the same commercial food free of antibacterials (Supra Profrango, Alisul Ltda.) throughout the experiment and water *ad libitum*. The yeasts were added in liquid state to commercial food using a Y industry mixer to guarantee the homogeneity of the feed.

#### Treatments

The control group (CON) was fed with non-supplemented feed; SBY group received feed supplemented with 1x10^7^ CFU g^-1^ of *Saccharomyces boulardii* (Floratil®, Merck) grown in Yeast Peptone Dextrose medium (YPD, Difco, USA); PPE group was fed with feed supplemented with 1x10^7^ CFU g^-1^ of *P*. *pastoris* X-33 (Invitrogen^®^, USA) grown in an effluent from the tanks of parboiling rice industry to which 15 g.L^-1^ of glycerol by-product from the biodiesel industry was added [19], and PPY group with feed supplemented with 1x10^7^ CFU g^-1^ of *P*. *pastoris* X-33 grown in YPD.

### Production of yeasts

The yeasts were grown at 28°C, 400 rpm and 1 vvm of sterile air for 24 hours in a New Brunswick 110 bioreactor (New Brunswick Scientific, NJ, USA) in volumes of 7 L of medium and 10% of inoculums. Pre-inoculums and inoculums were grown on an orbital shaker at 150 rpm and 28°C for 12 h, using YPD (Difco, USA) for those of SBY and PPY groups, and YM (Yeast Medium, Difco, USA) for PPE. 1M NaOH was used to adjust the pH of the medium in PPE to 5.5 at the beginning of the process. Two mL of antifoam Agripec 263 EA was diluted at a ratio of 1:5 in water and added to all the fermentations at the start of the experiment.

### Feed Convertion Ratio (FCR)

Each animal was weighed on days 1, 7, 14, 21, 28, 35 and 42 of the experiment. Weight gains were evaluated at 7, 14, 21, 28, 35 and 42 days by subtracting the weight of the animal at day one from that on the respective day. Feed conversions were estimated by dividing the weight gain of the group in the period assessed by the amount of feed consumed during the period. When deaths occurred, feed intake per animal was calculated, and the estimation of food intake corrected to the number of survivors of the respective group.

### Histology methodology

Tissue fragments of liver, spleen and intestines were fixed into formalin, dehydrated in increasing concentrations of ethanol and subsequently embedded in paraffin. Sections (3–5 μm thick) were cut using a rotary microtome (Leica RM 2255), mounted on glass slides and stained with hematoxylin and eosin. Histological examinations were performed using a light microscope (Nikon Eclipse 50-i).

### Titration of anti-IBD antibodies

Individual blood samples were collected from the wing vein of the animals on days 1, 14, 28 and 42 of the experiment, maintained at 37°C for 15 min and then at 4°C overnight. After centrifugation at 1800 *g* the serum was collected and stored at -20°C. Titration of anti-IBD antibodies by ELISA was performed by Mercolab Laboratory (Garibaldi, RS, Brazil) using IDEXX IBD Ab Test (Idexx Laboratories, Inc., Maine, USA) in accordance with the manufacturer's instructions.

### Safety

On day 42 of the experiment, ten animals from each group were euthanized (cervical dislocation) and necropsied for macroscopic evaluations of the liver, spleen, heart, bursa of Fabricius, proventriculus, small and large intestines. Sections of liver, spleen and intestines were stained with hematoxylin-eosin and evaluated by microscopy.

### Statistical analysis

ANOVA proc glm (general linear model of SAS) was used to analyze the data. Differences between the means were evaluated by the Duncan test (p<0.05) and the normality tested by Shapiro-Wilk.

## Results

### Feed efficiency

On the first day of the experiment there were no differences in the average weights between the groups. The supplemented groups SBY, PPE and PPY, had higher average weights throughout the experiment, with the exception of the weights at 35 days, when *P*. *pastoris* YPD had the lower value (Table 1). On days 7 and 14, the supplemented groups had significantly higher weights (p<0.05) than the control group. The effluent group (PPE) exhibited the highest average weights throughout the experiment (p<0.05).

**Table 1:** Average weight gain and feed conversion means of groups at 1, 7, 14, 21, 28, 35 and 42 days old.

Groups**	Weight gain (g)*						Feed conversion (kg)					
	7 days	14 days	21 days	28 days	35 days	42 days	7 days	14 days	21 days	28 days	35 days	42 days
CON	79 ±12^j^	239 ± 38^g^	531 ± 83^e^	929 ± 131^c^	1523 ± 170^b^	2178 ± 197^a^	1.43	1.69	1.86	2.03	1.85	1.89
SBY	86 ± 8^i^	266 ± 24^f^	564 ± 60^de^	964 ± 101^c^	1538 ± 146^b^	2181 ± 183^a^	1.31	1.53	1.78	1.83	1.89	1.92
PPE	94 ± 10^h^	275 ± 36^f^	577 ± 84^d^	983 ± 126^c^	1573 ± 166^b^	2197 ± 182^a^	1.32	1.87	1.95	1.98	1.98	1.99
PPY	92 ± 13^h^	272 ± 39^f^	559 ± 71^de^	951 ± 118^c^	1520 ± 165^b^	2184 ± 207^a^	1.42	1.64	1.82	1.82	1.67	1.73

*Different letter mean statistical difference (p<0,05) in weight gain ± standard deviation.

** CON–Control group; SBY–supplemented group with *Saccharomyces boulardii*; PPE—supplemented group with *P*. *pastoris* X-33 grown in industrial effluent; PPY—supplemented group with *P*. *pastoris* X33 grown in YPD.

On day 7 the *P*. *pastoris* groups (PPE and PPY) had significantly higher weight gains (p<0.05) while that of the *S*. *boulardii* group was higher than the controls (p<0.05). On day 14 there were no differences among the weight gain of the supplemented groups (p>0.05), although the weight gain of the control group was significantly lower (p<0.05). On day 21, only the animals in the effluent group (PPE) had significantly higher weight gains than those of the control group (Table 1).

Until day 21 the lower feed conversion was obtained by the *S*. *boulardii* group (SBY) (Table 1). On day 28, feed conversion of the *S*. *boulardii* (SBY) and *P*. *pastori* YPD (PPY) were not different (p<0.05). From the 28^th^ day until the end of the experiment the best feed conversion was obtained by the *P*. *pastoris* YPD group (Table 1).

### Anti-IBD titers

**Fig 1:**
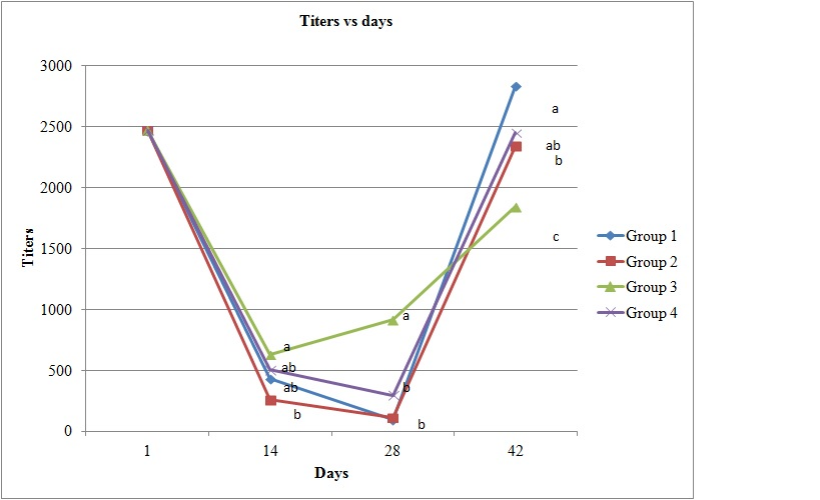
Mean titers of anti-IBD antibodies by ELISA. CON: Control; SBY: *S*. *boulardii*; PPE: *P*. *pastoris* effluent; PPY: *P*. *pastoris* YPD. Different letters signify the mean statistical differences on the day (p<0.05).

On day one all the chicks showed high titers (2500) of maternal antibodies against Infectious Bursal Disease. The titers fell steadily until day 28, with lower percentage of the chicks with titers above 1000 in the CON and SBY groups. After day 28 the titers of the animals in the four groups increased steadily surpassing that threshold (Table 2, Fig 1).

**Table 2:** Percentage of positive sera and sera with titers above 1000 of anti-IBD antibodies in chicks vaccinated at one day of age.

Groups	Positive sera (%)**			Sera with titers above 1000 (%)		
	14 days*	28 days*	42 days*	14 days	28 days	42 days
Control	46^b^	3^b^	100^a^	3	3	100
*S*. *boulardii*	42^a^	3^b^	100^a^	3	3	90
*P*. *pastoris* effluent	71^b^	64^a^	100^b^	17	45	90
*P*. *pastoris* YPD	62^b^	11^b^	100^b^	4	11	96

*Different letters mean statistical differences of mean titers of antibodies among the groups in the respective day (p<0,05).

** A day one positive sera and sera with titers above 1000 were 100%.

### Safety

**Fig 2:**
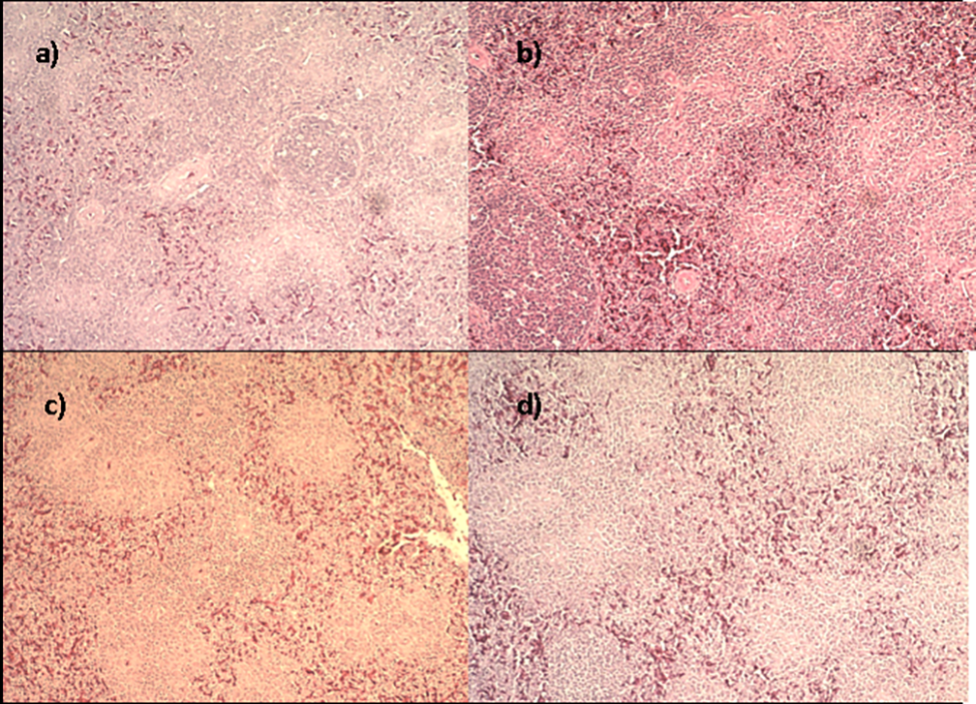
Histological study showing normal splenic tissue of groups (a) CON, (b) SBY, (c) PPE and (d) PPY. H&E (0bj 10x).

**Fig 3:**
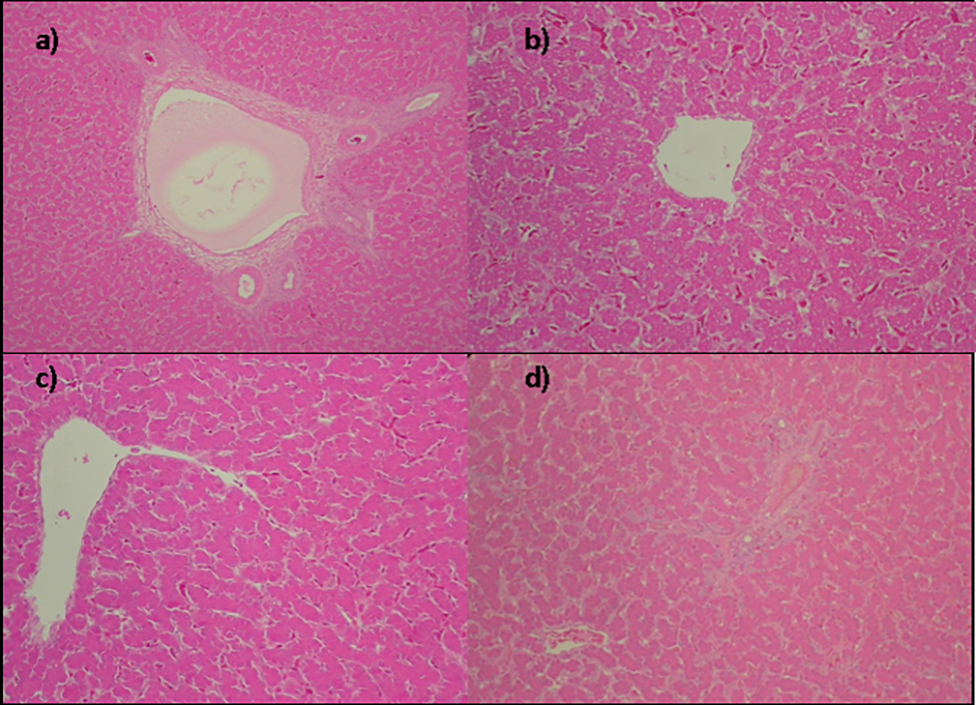
Histological study showing normal hepatocyte and portal vein of the groups (a) CON, (b) SBY, (c) PPE and (d) PPY. H&E (0bj 10x).

No significant macroscopic changes in liver, spleen, heart, bursa of Fabricius, proventriculus, small and large intestine were found in samples collected from the groups. In the groups with higher weight gains no signs of degenerative or inflammatory lesions were found in the organs evaluated by histopathology (Figs 2 and 3).

## Discussion

*Pichia pastoris* is a yeast widely used as vector for the expression of heterologous proteins. Although information about its utilization for other purposes is scarce, Gil de los Santos et al. [4] showed that it enhances weight gains in broiler chickens. In a previous paper [19] our group showed that *P*. *pastoris* X-33 grown in parboiled rice effluent supplemented with glycerol of the biodiesel industry, two industrial wastes without commercial value was able to reduce 38.8% of COD (Chemical Oxygen Demand), 75% NTK—N and 90% P in the effluents of the parboiled rice industry. This paper reports for the first time the probiotic properties in broilers of this yeast grown in the effluents of that industry.

Among the great variety of microorganisms used as probiotics, yeasts are of special interest due to their capacity to grow in low-cost media and even in residues or effluents that constitute environmental hazards, and for its capacity to survive in the conditions of animal breeding premises and incorporated to animal feed. Research has extensively shown a great variation in the results obtained, even with the same probiotic and the same animal species, demonstrating that different experimental conditions may affect how yeasts behave as probiotics. In the experiment here reported the properties of *P*. *pastoris* X-33 grown in an effluent of the parboiled rice industry supplemented with biodiesel glycerol were compared in identical experimental conditions with other probiotics produced by our group.

Our results showed that during the first three weeks body weight gains were higher in the supplemented groups, although those supplemented with *S*. *boulardii* and *P*. *pastoris* grown in YPD were not significantly higher than the controls. Salim et al. [23] demonstrated that a mixture of microorganisms containing *Saccharomyces cerevisiae* increased the body weight gains of broiler chickens at 21 days of age while Bai et al. [24] also reported that it improved feed efficiency in animals fed during the first 21 days of life, although there were no significant differences between supplemented and non-supplemented animals after that period. Aluwong et al. [25] found that probiotics reestablished the conditions of eubiosis in the digestive tract during the first three weeks and that they could increase feed efficiency after that period.

Our results at slaughter (42 days) showed that, although there were no statistical differences between the treatments, weight gains in the groups that received *P*. *pastoris* grown in effluent (PPE), *P*. *pastoris* grown in YPD (PPY) and *S*. *boulardii* (SBY) were higher than the control (0.9%, 0.2%, and 0.1%, respectively). These results are below the differences of 8% obtained with *S*. *boulardii* [3], 5.7% with *Clostridium butyricum* and 2.8% with *Enterococcus faecium* [26]. These differences may be due to the conditions of the experiments, generally performed in controlled environments. Several researchers on probiotics agree that the real effects of probiotics are shown under field conditions where the probiotics produce significant differences in the parameters evaluated. In terms of feed conversions at 42 days, our results showed that animals supplemented with *P*. *pastoris* grown in YPD (PPY) consumed 8.47% less feed than the control group to achieve the same live weight. Similar results were found by Gil de los Santos et al. [4] in broiler chickens fed with *P*. *pastoris* KM71H, which consumed 6.6% less feed than the controls, and Mookiah et al. [27] in broiler chickens fed a multistrain *Lactobacillus* probiotic, which consumed 6.8% less feed than the nonsupplemented group.

Another important property of probiotics is their capacity to modulate immune responses. Some experiments showed that probiotics have immunomodulatory effects depending on the characteristics of the immunogen [28–30] and the adjuvant used in the vaccines [31]. The effect of probiotics on the enhancement of the titers of specific and natural antibodies in the serum and gut of chickens against the tetanus toxin was already reported [32, 33]. Gil de los Santos et al. [4] demonstrated that *P*. *pastoris* KM71H and its recombinant construct containing the gene of the alpha toxin of *Clostridium perfringens* increased seroconversion against alpha toxin of *Clostridium perfringens* in supplemented animals.

Infectious Bursal Disease (IBD) is one of the more economically significant diseases that affect commercially produced chickens worldwide [34]. For this reason, in this study we monitored the dynamics of the immune response elicited in broilers supplemented with our probiotics by an extensively used IBD vaccine in Brazil. The control of this disease in broilers is based on the passive immunity conferred by maternal yolk antibodies [35] followed by active immunity induced by several types of vaccines administered on the first day of age or later. In our experiment we detected decreasing titers of IBD antibodies in the control (CON), *S*. *boulardii* (SBY) and *P*. *pastoris* (PPY) grown in YPD groups (Fig 1) during the period of passive immunity. Knoblich et al. [36] reported a similar observation with the extinction of IBD detectable antibodies by ELISA 28 days after vaccination in chicks vaccinated at one day of age against a bivalent IBD-Marek's disease vaccine. In our experiment, only 3% of the chicks in the control group showed antibodies against IBD four weeks after vaccination, whereas on the same date, 64% of those of the *P*. *pastoris* effluent group (PPE) had antibodies with a mean titre of 913, showing also the highest percentages of immune animals at 14 and 28 days of age (Fig 1), suggesting that the probiotic has an immunomodulatory effect, maintaining a high level of maternal immunity during the period in which the active immunization induced by the vaccine is being established. At 42 days, the mean titer of the *P*. *pastoris* effluent group (PPE) (1842) was significantly lower than those detected in the control (2831), *P*. *pastoris* YPD (2448), and *S*. *boulardii* (2340) groups, suggesting that the higher antibody titers present in previous periods could have interfered in the vaccinal virus replication, resulting in a reduction in the final mean titer of this group, similar to that observed in other species when active immunization is initiated during the period of passive maternal immunity. Even so, the titers were compatible with adequate immunity.

The *P*. *pastoris* effluent group (PPE) maintained the higher titre of antibodies until 35 days of age, in contrast with the lower titers observed in the other groups on days 14 and 28. Although the study of the cause of these observations was without the objective of our experiment, it could be argued that this effect could be due to the differences in protein expression by this yeast due to pH and medium composition that, as has been already shown, could modify the composition of the yeast’s cell membrane and, thus, its interaction with intestinal cells [37]. Recent studies used molecular dynamics simulations suggest that components of medium could induce beneficial interaction between the probiotic and target cells [38]. Choi and Park [14] demonstrated that the composition of proteins and lipids of *Pichia guilliermondii* A9 differed when it was grown in a residue of the fermentation of kimchi or in yeast medium. Deepika et al. [39] also showed that the temperature of growth and pH of the medium influenced surface composition, hydrophobicity, and the adhesion of *Lactobacillus rhamnosus* to Caco-2 cells.

The administration of different probiotics from the second day of life onwards could have produced modifications in the gut’s microbiota and the expression of different genes [40]. The higher weight gain obtained by the group supplemented with *P*. *pastoris* grown in the effluent medium (PPE) could also have favored the maturation of the immune system improving seroconversions. The maturation of the immune system in chickens begins between the 2^nd^ and 4^th^ day of life and is directly correlated with intestinal microbiota alterations until the 19^th^ day [41].

Our results showed that *P*. *pastoris* X-33 grown in an effluent from the parboiled rice industry supplemented with glycerol byproduct from the biodiesel production process had beneficial immunomodulatory effects in broiler chickens. The group supplemented with this yeast had the higher percentage of animals with high titers of anti-IBD antibodies during the critical passive immunity period and the beginning of the active immunity period, when compared with the control group or those supplemented with the other two probiotics tested.

Previous work done by our group [19] showed that *Pichia pastoris* X-33 grown in effluents of the parboiled rice industry supplemented with glycerol of biodiesel reduces the environmental impacts of the effluent. Furthermore, according to our results, the yeast has probiotic properties that could be used in the breeding of broiler chickens, adding value to effluents.

As far as we know no other experiments of validation of the probiotic properties for poultry of *P*. *pastoris* that eliminated pollutants from effluents of the rice parboiling industry have been published, making comparisons impossible.

We concluded that *Pichia pastoris* X-33 grown in the effluent of the parboiled rice industry supplemented with biodiesel glycerol can be used as a probiotic for broiler chickens, improving the response to IBD vaccines and, when grown in YPD, increasing feed efficiency.
